# Disentangling the divergent causal pathways underlying the association between body mass index and bone mineral density: a comprehensive Mendelian randomization study

**DOI:** 10.1186/s12916-025-04139-2

**Published:** 2025-05-28

**Authors:** Xunying Zhao, Lin He, Xueyao Wu, Li Zhang, Jinyu Xiao, Changfeng Xiao, Yang Qu, Jingwei Zhu, Chenjiarui Qin, Deqin Huang, Pengyue Shen, Tao Han, Mengyu Fan, Jiayuan Li, Stephen Burgess, Xia Jiang

**Affiliations:** 1 Department of Epidemiology and Health Statistics and West China Institute of Preventive and Medical Integration for Major Diseases, West China School of Public Health and West China Fourth Hospital, Sichuan University, Chengdu, Sichuan China; 2 Longquanyi District of Chengdu Maternity and Child Care Health Hospital, Chengdu, Sichuan China; 3 Division of Cancer Epidemiology and Genetics, National Cancer Institute, Rockville, Maryland USA; 4 Department of Nutrition and Food Hygiene, West China School of Public Health and West China Fourth Hospital, Sichuan University, Chengdu, West China China; 5 MRC Biostatistics Unit, Cambridge Institute of Public Health, University of Cambridge, Cambridge, UK; 6 Department of Clinical Neuroscience, Center for Molecular Medicine, Karolinska Institutet, Solna, Stockholm, Sweden

**Keywords:** Body mass index, Estimated bone mineral density, Mendelian randomization, Divergent causal pathways, Causal inference

## Abstract

**Background:**

While the protective role of body mass index (BMI) in bone mass has been well-documented, the divergent associations between BMI and estimated bone mineral density (eBMD), attributed to its highly heterogeneous nature, remain insufficiently understood.

**Methods:**

Leveraging the hitherto largest genome-wide summary statistics, we conducted a two-sample Mendelian randomization (MR) to re-evaluate the effect of genetically predicted BMI on eBMD. Then, MR-Clust was applied to examine the potential presence of distinct causal pathways underlying the BMI-eBMD link. Utilizing tissue-partitioned MR, we estimated the distinct effects of separated tissue-specific subcomponents of BMI on eBMD, further supplemented by multivariable MR of body composition phenotypes on eBMD.

**Results:**

We reconfirmed the significant positive association between genetically predicted BMI and eBMD (*β*_IVW_ = 0.13, *P* value = 1.28 × 10^−34^). Potential distinct causal pathways contributing to the observed total effect were identified by MR-Clust, with some exerting a protective effect while others leading to its deterioration. Tissue-partitioned MR suggested a marginally independent protective association between skeletal muscle-tissue instrumented BMI and eBMD (*β*_IVW_ = 0.14, *P* value = 4.98 × 10^−2^) after accounting for adipose-tissue instrumented BMI, which was supported by the independent association between genetically predicted lean mass and eBMD after accounting for other body composition phenotypes.

**Conclusions:**

Our results shed preliminary insights into the intricate relationship between obesity and bone mass, highlighting divergent causal pathways underlying the association between BMI and eBMD. Our findings emphasize the potential importance of precision obesity management over merely a general indicator as BMI in future public health strategies for osteoporosis prevention.

**Supplementary Information:**

The online version contains supplementary material available at 10.1186/s12916-025-04139-2.

## Background

The relationship between body mass index (BMI) and osteoporosis has been a topic of debate and research focus in recent years. While BMI is widely recognized as a risk factor for multiple bone-related diseases (e.g., osteoarthritis, rheumatoid arthritis, and gout) [1–3], extensive research has revealed its unexpected protective role in bone mass, termed the “obesity paradox” [4–7]. Two meta-analyses have consistently reported positive associations between BMI and bone mineral density (BMD) at multiple sites, with obese individuals having 0.05 ~ 0.11 g/cm^2^ higher BMD than non-obese individuals [8, 9]. These associations have been further validated through Mendelian randomization (MR) studies, which show that genetically predicted BMI positively associates with estimated BMD (eBMD) (*β* = 0.12 ~ 0.13 per 1-standard deviation [SD]) [10–12]. These unexpected findings underscore the complex interplay between BMI and bone mass, yet its underlying mechanisms remain incompletely understood.

The complexity in the relationship between BMI and bone mass may arise from the heterogeneous nature of BMI itself, as it is a highly complex trait that encompasses multiple phenotypes [13, 14]. Individuals with higher BMI tend to exhibit greater lean mass (LM), which can enhance bone mass through mechanical loading and osteoblast activation [15, 16]. However, higher BMI is also associated with an excessive accumulation of fat mass (FM), which may promote osteoclast differentiation and elevate bone resorption through inflammatory and endocrine pathways [17]. Additionally, subcutaneous and visceral adipose tissues, both strongly linked to BMI, have distinct structures, functions, and physiological consequences [18], potentially exerting differing influences on bone mass [19]. Therefore, the observed impact of BMI on bone mass may represent a combination of effects from different factors, including both the “favorable” and “unfavorable” types of body compositions [13, 14]. Despite this complexity, previous studies have primarily focused on the aggregated effect of BMI, without disentangling the effects of specific compositions or determining which composition plays a predominant role.

Recent advances in methodological techniques and genetic research provide an opportunity to dissect the complex relationship from a genetic aspect. Two newly developed MR extensions—MR-Clust and tissue-partitioned MR—enable us to identify distinct causal pathways by clustering genetic variants based on their effects [20] and the partitioning of BMI effects through tissue-specific gene expression [21, 22], respectively. Furthermore, a large-scale genome-wide association study (GWAS) of BMI (*N* = 806,834) has identified substantially more genetic variants than ever before, greatly enhancing the statistical power [23]. These advances offer new possibilities for understanding how different components of BMI influence bone mass.

Therefore, the current study aimed (i) to re-evaluate the effect of genetically predicted BMI on eBMD by leveraging the hitherto largest GWAS summary data available for the two traits; (ii) to examine the presence of distinct causal pathways underlying the BMI-eBMD link by using MR-Clust; (iii) to estimate the distinct effects of tissue-partitioned components of BMI on eBMD by using tissue-partitioned MR; and (iv) to complement the tissue-partitioned MR using genetic variation data of four body composition phenotypes, including LM, FM, body fat percentage (BFP), and body weight (BW).

## Methods

**Fig. 1:**
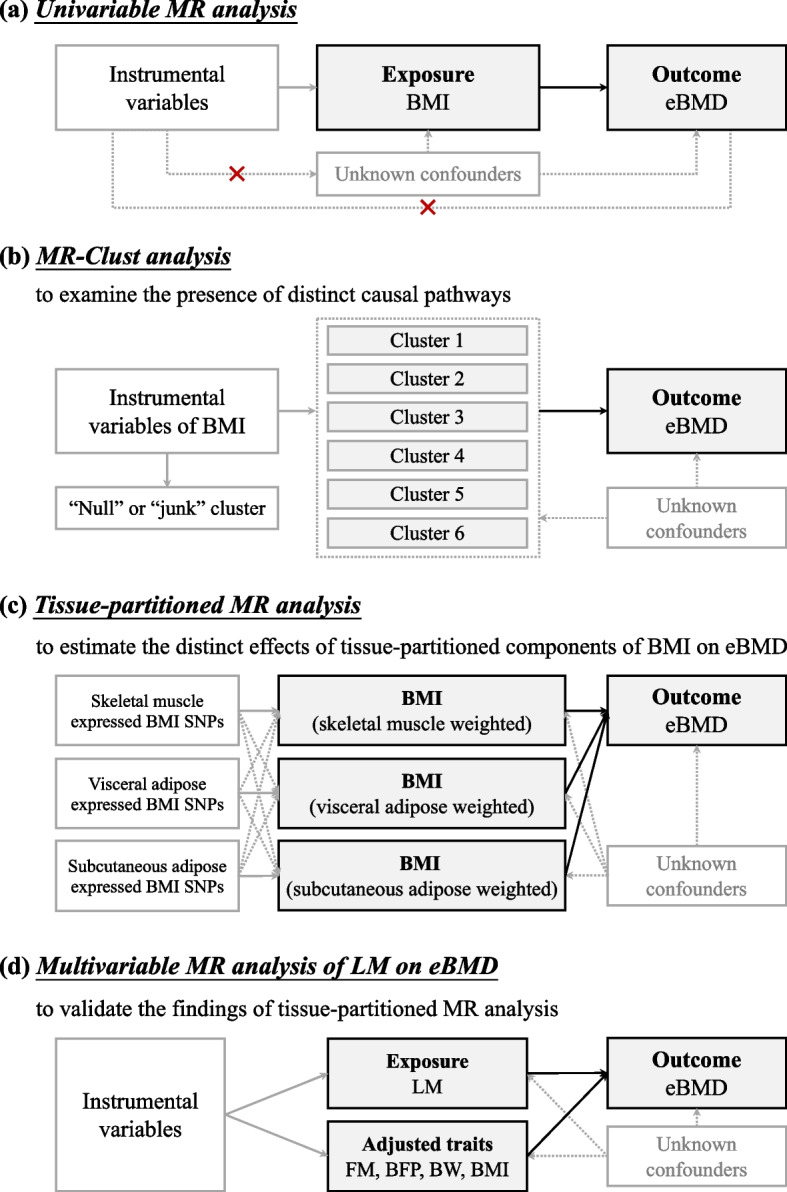
Overall design of this study. BMI, body mass index; eBMD, estimated bone mineral density; LM, lean mass; FM, fat mass; BFP, body fat percentage; BW, body weight; MR, Mendelian randomization; SNP, single nucleotide polymorphism

This study adhered to the guidelines of the Strengthening the Reporting of Observational Studies in Epidemiology—Mendelian Randomization (STROBE-MR) (Additional file 1, https://www.strobe-mr.org/) [24, 25]. The summary-level data utilized in our study were publicly available, all of which had received ethical approval in the original studies. The overall study design is shown in Fig. 1.

### Data source

#### GWAS datasets

We obtained the largest GWAS summary statistics for BMI from a meta-analysis of the UK Biobank (UKB) and the Genetic Investigation of Anthropometric Traits (GIANT) consortium, involving 806,834 individuals of European ancestry [23, 26]. We obtained the largest GWAS summary statistics for eBMD, measured by heel quantitative ultrasound speed of sound and broadband ultrasound attenuation [27, 28], which involves 426,824 individuals of European ancestry from the UKB.

For the four complementary body composition phenotypes, GWAS summary statistics of LM were obtained from the Neale Lab consortium, based on 331,291 European individuals [29, 30]. GWAS summary statistics of FM were acquired from the MRC-IEU consortium, involving 454,137 European individuals [29, 31]. GWAS summary statistics of BFP were acquired from UKB, comprising 155,961 European individuals [32, 33]. GWAS summary statistics of BW were acquired from a meta-analysis of UKB and Biobank Japan (BBJ), including 525,523 individuals (~ 69% of European ancestry) [34, 35]. Detailed information regarding each GWAS dataset was provided in Additional file 2: Table S1.

#### Tissue-specific gene expression datasets

We utilized expression quantitative trait loci (eQTL) data from GTEx v8 (Genotype-Tissue Expression, version 8, ~ 80% European ancestry) [36]. Given the intricate roles of LM and FM in bone mass, we hypothesized that genes associated with BMI expressed in skeletal muscle (the main component of LM) may drive the effect by promoting bone growth [15, 16], while those expressed in subcutaneous or visceral adipose tissues (the main components of FM) may be inclined to disturb bone metabolism through pathways such as oxidative stress and inflammation [17]. Accordingly, we focused on three tissues—the skeletal muscle (*N* = 706), the visceral adipose (*N* = 469), and the subcutaneous adipose (*N* = 581)—in our tissue-partitioned analysis. Data was downloaded from the Summary Mendelian Randomization website (https://cnsgenomics.com/software/smr/), which was mapped to hg19 genome build using the GRCh37 reference assembly [37, 38].

### Statistical analysis

#### Univariable MR analysis of BMI on eBMD

A univariable MR analysis was first performed to investigate the total effect of genetically predicted BMI on eBMD, as shown in Fig. 1a. To avoid missing association signals due to the stringent *P* value threshold applied by the original GWAS (5.0 × 10^−9^), we employed a standard clumping strategy to re-screen instrumental variables (IVs) under genome-wide significance (5.0 × 10^−8^) using PLINK’s “clumping” function (parameters: –clump-p1 5e-8 –clump-p2 1e-5 –clump-r2 0.05 –clump-kb 5000) [39]. If an instrumented single nucleotide polymorphism (SNP) was unavailable in the eBMD GWAS dataset, we used LDlinkR package in R to identify a proxy SNP with linkage disequilibrium (LD) ${r}^{2}$ > 0.8 to minimize the exclusion of IVs [40]. We computed the proportion of trait variance explained (*R*^2^) and the *F*-statistic for each IV, considering an *F*-statistic greater than 10 as evidence of a robust IV [41]. The statistical power of our analysis was assessed utilizing a web-based application (https://sb452.shinyapps.io/power/) [42].

Inverse variance weighted (IVW) method was applied as our primary approach, which combines estimates from each IV to generate an overall MR estimate, assuming the validity of all IVs [43]. The estimates of *β* represent the effect size, indicating the average increase in eBMD (measured in SD = 0.14 g/cm^2^) associated with each 5-kg/m^2^ increase in genetically predicted BMI. Cochran’s *Q* statistics were utilized to assess the global heterogeneity of IVW [44]. We conducted several sensitivity analyses to validate the IV assumptions of MR, including (i) MR-Egger regression [45], (ii) weighted median [46], (iii) IVW after excluding pleiotropic IVs (accessed in GWAS catalog on 04/08/2024, https://www.ebi.ac.uk/gwas/), (iv) IVW after excluding palindromic IVs, (v) leave-one-out analysis to identify outliers that might bias the MR estimates [47], and (vi) MR-PRESSO (Mendelian Randomization Pleiotropy Residual Sum and Outlier) to detect and correct the potential horizontal pleiotropy [48]. We also repeated IVW by using BMI-associated SNPs reported from the original GWAS (*P* value < 5.0 × 10^−9^) to compare and demonstrate the reliability of our expanded set of IVs for BMI.

An MR estimate was considered significant if the *P* value was < 0.05 in IVW and showed directional consistency across all sensitivity analyses.

#### MR-Clust analysis of BMI on eBMD

Given the high-heterogeneous nature of BMI, we scrutinized potential divergent causal pathways between BMI and eBMD by first performing an MR-Clust analysis [20], as shown in Fig. 1b. MR-Clust serves as a tool for clustering genetic variants based on their causal estimates derived from MR analysis. Under assumptions of linearity and homogeneity, this method delineates distinct substantive clusters representing local average causal associations, with all genetic variants within a cluster exerting similar effects on the outcome. The optimal number of substantive clusters is determined automatically by the algorithm using Bayesian information criterion, which balances model fit against complexity to avoid overparameterization. MR-Clust also introduces the concept of the “null” cluster for non-causal variants and the “junk” cluster for variants whose estimates do not fit into any substantive clusters, thereby minimizing false-positive findings and enhancing the credibility of the substantive clusters. To avoid chance similarity of causal estimates from different variants, we applied this method by assigning variants to a cluster only if the conditional probability of cluster assignment was ≥ 0.8, and reporting a cluster only when at least four variants met this criterion.

To uncover heterogeneous biological insights for SNPs in clusters with different effects, we performed detailed functional annotation using the Ensembl Variant Effect Predictor (VEP) [49] and then assessed gene enrichment through the WebGestalt tool, focusing on Gene Ontology (GO) biological processes and Kyoto Encyclopedia of Genes and Genomes (KEGG) pathways [50]. Clusters were combined into positive and negative effect groups in enrichment analysis.

#### Tissue-partitioned MR analysis of BMI on eBMD

We proceeded to utilize tissue-partitioned MR to disentangle the phenotypic subcomponents of BMI and investigate which subcomponent predominantly contributes to the observed overall causal association, as shown in Fig. 1c. In brief, tissue-partitioned MR discerns genetic variants associated with BMI into tissue-specific sets (proxying the certain-tissue instrumented BMI) based on whether they colocalize with gene expression in this tissue type.

To separate the IVs of BMI into tissue-specific subsets, we employed the Bayesian method “coloc” [51] to investigate whether the genetic variants associated with BMI at each genome-wide locus (encompassing all SNPs within a 200-kb window) also act as causal eQTLs for a nearby gene in the three tissue types of interest. Variants within the major histocompatibility complex region (chr6: 24,000,000 ~ 36,000,000) were excluded. A Posterior Probability of Hypothesis 4 (PPH4) > 0.8 indicates strong evidence of colocalization, with a more relaxed threshold of PPH4 > 0.7 used to examine the robustness of the primary results. Ultimately, the total effect of BMI was separated into three phenotypic subcomponents according to the subsets of IVs, referred to as skeletal muscle-tissue instrumented BMI, visceral adipose-tissue instrumented BMI, and subcutaneous adipose-tissue instrumented BMI.

Utilizing the three subsets of tissue-specific IVs, we first estimated the unadjusted effect of each BMI phenotypic subcomponent on eBMD by univariable MR. To investigate which tissue-specific subcomponent predominantly drives the total effect, we further conducted multivariable MR to evaluate the independent effect of each phenotypic subcomponent of BMI on eBMD after accounting for the effects of the other subcomponents. We incorporated each two of the three tissue-instrumented BMI in a multivariable model pairwise, with the beta estimates of each IV weighted by its tissue-specific PPH4 value [52]. Composite IVs of the phenotypic subcomponents of BMI were used in multivariable settings.

#### Supplemental MR analysis of LM on eBMD

As a marginally independent effect of skeletal muscle-instrumented BMI on eBMD was identified in tissue-partitioned MR analysis, we conducted a supplemental MR by treating LM as the exposure and eBMD as the outcome to validate the potentially predominant role of skeletal muscle-related causal pathway, as shown in Fig. 1d. We used the same parameters mentioned above to clump independent trait-associated SNPs as IVs. We first performed a univariable MR analysis to evaluate the total effect of LM on eBMD. Following this, we carried out multivariable MR [52] analyses to estimate the independent effect of LM on eBMD while taking into account potential confounding effects of FM, BFP, BMI, and BW, sequentially. The estimates of *β* represent the effect size, indicating the average increase in eBMD (measured in SD = 0.14 g/cm^2^) associated with each 5-kg increase in genetically predicted LM, FM, and BW, and each 1-SD (8.2%) increase in genetically predicted BFP. Composite IVs of body composition phenotypes were used in the multivariable MR. We pruned SNPs in LD with *r*^2^ ≥ 0.05 to minimize redundancy and correlation in the pooled set of IVs.

#### Sensitivity analysis

As both the GWAS of BMI and eBMD included UKB data, we conducted a sensitivity analysis using an alternative BMI GWAS from Locke et al. (BMI-GWAS [2015]) with 322,154 individuals of European descent [53], which does not include UKB data. We repeated our main analysis using the effect sizes and standard errors from this non-overlapping dataset.

All analyses were performed in R software (v. 4.2.2), utilizing various packages including “TwoSampleMR” (v. 0.5.6), “MendelianRandomization” (v. 0.7.0), “LDlinkR” (v. 1.3.0.9), “MRPRESSO” (v. 1.0), “mrclust” (v. 0.1.0), and “coloc” (v. 5.1.0).

## Results

### Univariable MR analysis

A total of 1424 independent BMI-associated SNPs were determined as IVs, all of which exhibited minimal likelihood of weak instrument bias (*F*-statistics ≥ 28.26, Additional file 2: Table S2). We have 80% power to detect an effect of genetically predicted BMI on eBMD with a minimum effect size (*β*) of 0.01 (per 5-kg/m^2^ increase) at a significance level of 0.05.

**Fig. 2:**
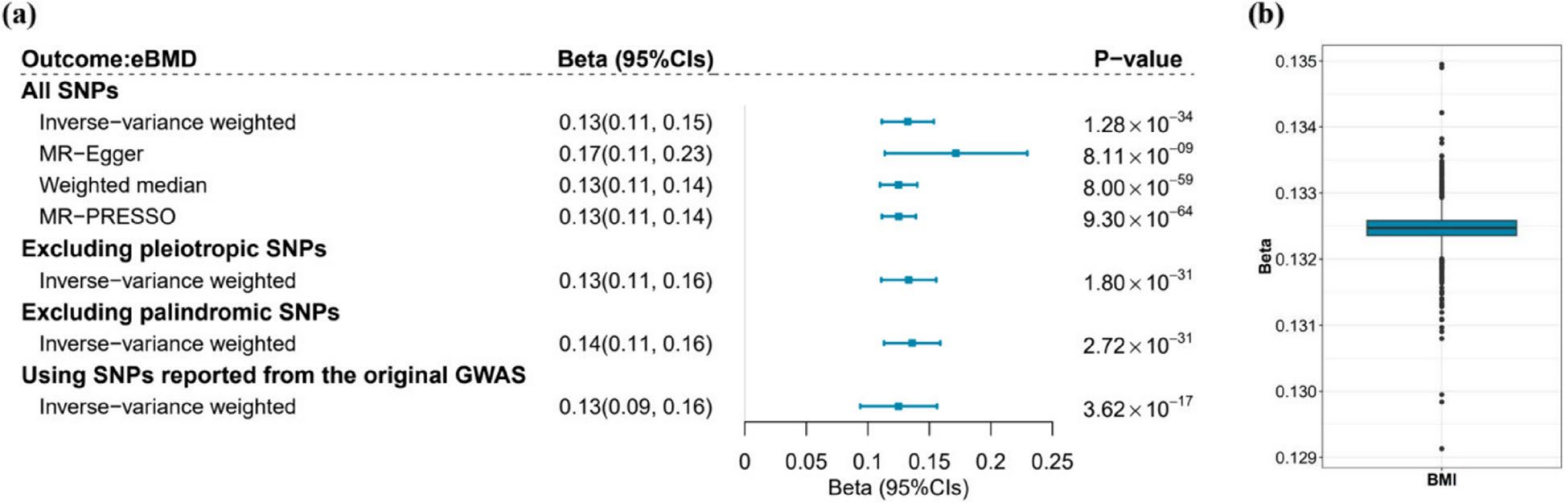
Results of univariable Mendelian randomization analysis conducted for BMI and eBMD. **a** Forest plot summarizing the results of univariable Mendelian randomization (MR) analysis. Blue squares represent point estimates; horizontal bars represent 95% confidence intervals. **b** Box plot representing the results from leave-one-out analysis which excluded each single nucleotide polymorphism at a time to estimate the effect of BMI on eBMD. The estimates of *β* are expressed per 5-kg/m^2^ increase in genetically predicted BMI. The upper, middle, and lower lines depict the upper quartile, median, and lower quartile of beta values, respectively. The lines extending from the top and bottom of the box indicate the maximum and minimum values. Outliers are represented by scatter points. BMI, body mass index; eBMD, estimated bone mineral density; SNP, single nucleotide polymorphism; GWAS, genome-wide association study

As shown in Fig. 2, we observed a significant protective association between genetically predicted BMI and eBMD (*β*_IVW_ = 0.13, 95% confidence intervals [95%CIs] = 0.11 ~ 0.15, *P* value = 1.28 × 10^−34^), with heterogeneity indicated across individual SNP estimates (*P*_Cochran’s*Q*_ < 0.05, Additional file 2: Table S3). The findings of IVW were statistically significant and directionally consistent across all sensitivity analyses. MR-Egger regression (*β*_MR-Egger_ = 0.17, 95%CIs = 0.11 ~ 0.23, *P* value = 8.11 × 10^−9^) and weighted median approach (*β*_weighted median_ = 0.13, 95%CIs = 0.11 ~ 0.14, *P* value = 8.00 × 10^−59^) largely supported the results derived from IVW. After removing 198 pleiotropic SNPs (*β*_IVW_ = 0.13, 95%CIs = 0.11 ~ 0.16, *P* value = 1.80 × 10^−31^) or 171 palindromic SNPs (*β*_IVW_ = 0.14, 95%CIs = 0.11 ~ 0.16, *P* value = 2.72 × 10^−31^), the results of IVW remain substantially unaltered. The leave-one-out and MR-PRESSO analyses showed that the observed MR estimates were not influenced by any outlying variant. We also observed a consistent protective association between genetically predicted BMI and eBMD when using IVs reported from the original GWAS, validating the reliability of the re-clumped IVs for BMI (*β*_IVW_ = 0.13, 95%CIs = 0.09 ~ 0.16,* P* value = 3.62 × 10^−17^, Fig. 2).

### MR-Clust analysis

**Fig. 3:**
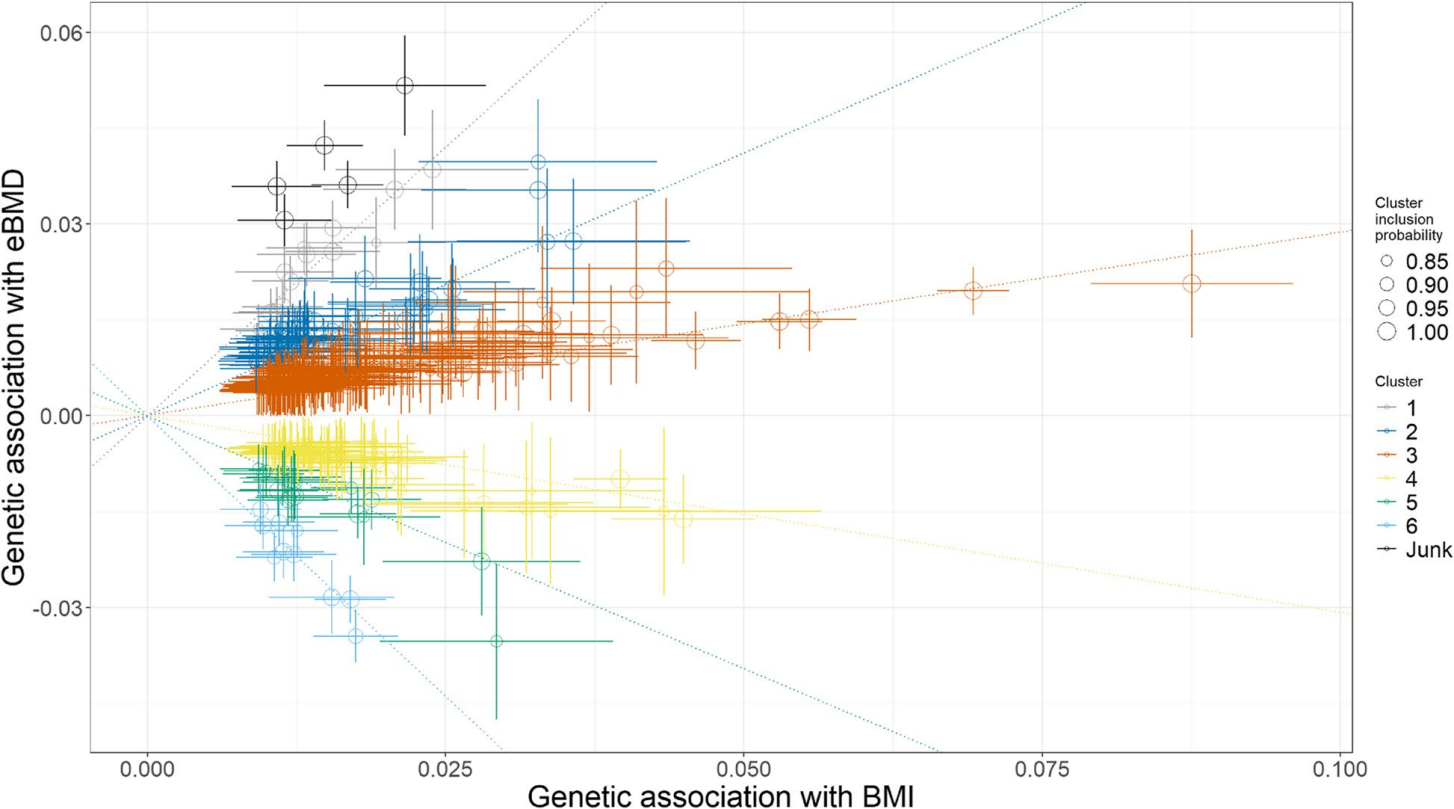
Results of MR-Clust showing clustered heterogeneity in Mendelian randomization analysis for the causal effects of BMI on eBMD. Variants are only assigned to a cluster if the conditional probability is ≥ 0.8, and clusters are only displayed if at least four variants are assigned to the cluster. Each genetic variant is represented by a point. Error bars are 95% confidence intervals for the genetic associations. Colors represent the clusters, and dotted lines represent the cluster means. BMI, body mass index; eBMD, estimated bone mineral density

We continued to evaluate the potential divergent causal pathways underlying BMI and eBMD by using MR-Clust building upon the robust association observed in univariable MR. Following stringent criteria for variant number and conditional probability, the genetic variants of BMI were classified into six substantive clusters (Additional file 2: Table S4). Clusters 1–3, consisting of 13, 63, and 227 variants respectively, continued to suggest positive effects on eBMD, while clusters 4–6, comprising 68, 19, and 10 variants, demonstrated negative effects (Fig. 3 and Additional file 2: Table S5). There was no heterogeneity observed within each cluster (all *P*_Cochran’s*Q*_ > 0.05).

Distinct biological pathways were identified through enrichment analysis, with SNPs in positive effect cluster (clusters 1–3) more related to neuronal processes and SNPs in negative effect cluster (clusters 4–6) more involved in cell growth and developmental regulation (Additional file 2: Table S6). After multiple testing corrections (Benjamini-Hochberg), GO analysis revealed that SNPs in clusters 1–3 were significantly enriched in axon development (GO:0061564), while the SNPs in clusters 4–6 showed enrichment in cell growth (GO:0016049), regulation of cell morphogenesis (GO:0022604), regulation of neuron projection development (GO:0010975), regulation of anatomical structure size (GO:0090066), and axon development (GO:0061564). No pathways survived multiple testing corrections in KEGG analysis.

### Tissue-partitioned MR analysis

Due to the evidence for divergent pathways underlying the relationship between BMI and eBMD from MR-Clust, tissue-partitioned MR analysis was further conducted to estimate the independent causal associations between distinct BMI subcomponents and eBMD. After conducting colocalization analysis, we identified 230, 218, and 236 SNPs for skeletal muscle-, visceral adipose-, and subcutaneous adipose-tissue instrumented BMI, respectively (Additional file 2: Tables S7–S9). The average effect sizes of tissue-partitioned instruments for BMI are comparable (Additional file 2: Tables S10–S11).

**Fig. 4:**
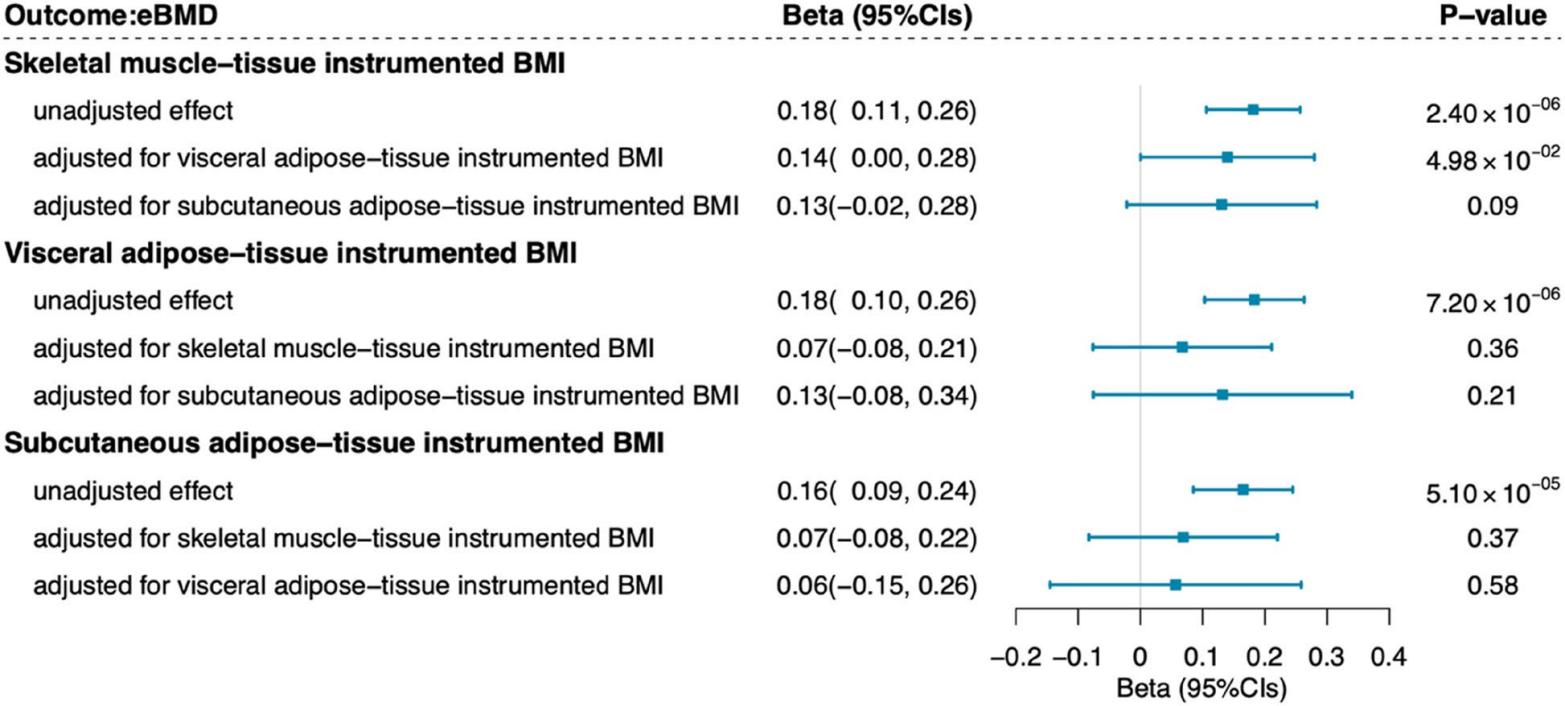
Results of tissue-partitioned Mendelian randomization analysis conducted for BMI and eBMD. Univariable Mendelian randomization (MR) analysis estimated the unadjusted effect of subcutaneous adipose-, visceral adipose-, and skeletal muscle-tissue-instrumented BMI on eBMD separately. Multivariable MR analysis estimated the independent effect of each phenotypic subcomponent of BMI after accounting for the effects of the other subcomponents. Blue squares represent point estimates; horizontal bars represent 95% confidence intervals. BMI, body mass index; eBMD, estimated bone mineral density; SNP, single nucleotide polymorphism

We identified evidence suggesting unadjusted protective effects of skeletal muscle-tissue instrumented BMI (*β*_IVW_ = 0.18, 95%CIs = 0.11 ~ 0.26, *P* value = 2.40 × 10^−6^), visceral adipose-tissue instrumented BMI (*β*_IVW_ = 0.18, 95%CIs = 0.10 ~ 0.26, *P* value = 7.20 × 10^−6^), and subcutaneous adipose-tissue instrumented BMI (*β*_IVW_ = 0.16, 95%CIs = 0.09 ~ 0.24, *P* value = 5.10 × 10^−5^) on eBMD in univariable MR (Fig. 4). However, the associations between visceral adipose-tissue instrumented BMI, subcutaneous adipose-tissue instrumented BMI, and eBMD attenuated to null in multivariable MR after adjusting for the other two phenotypic subcomponents of BMI separately. Notably, the protective association between skeletal muscle-tissue instrumented BMI and eBMD remained marginally significant and attenuated slightly when adjusting for visceral adipose-tissue instrumented BMI (*β*_IVW_ = 0.14, 95%CIs = 0.00 ~ 0.28, *P* value = 4.98 × 10^−2^, Fig. 4). The findings were highly consistent in the sensitivity analysis with an expanded set of IVs using a more relaxed threshold of PPH4 > 0.7 (Additional file 2: Table S12).

### Supplemental MR analysis

**Fig. 5:**
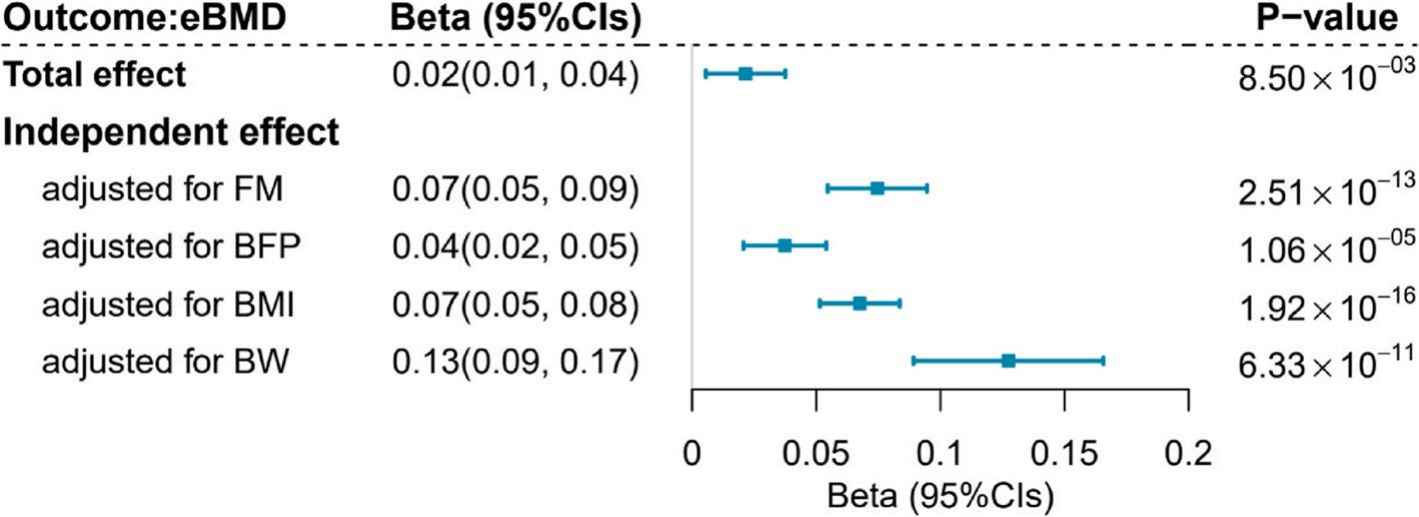
Results of univariable and multivariable Mendelian randomization analysis conducted to estimate the total effect and independent effect of lean mass on eBMD. Blue squares represent point estimates; horizontal bars represent 95% confidence intervals. The estimates of *β* are expressed per 5-kg increase in genetically predicted LM. eBMD, estimated bone mineral density; SNP, single nucleotide polymorphism; FM, fat mass; BFP, body fat percentage; BMI, body mass index; BW, body weight

To validate whether the skeletal muscle pathway is independently responsible for driving the effect of BMI on eBMD, we utilized GWAS data on body composition phenotypes to conduct a supplemental MR. We obtained 981 LM-associated SNPs as IVs, alongside 917 for FM, 92 for BFP, and 992 for BW. Univariable MR showed an unadjusted protective association between genetically predicted LM and eBMD (*β*_IVW_ = 0.02, 95%CIs = 0.01 ~ 0.04, *P* value = 8.50 × 10^−3^). Multivariable MR further demonstrated that this protective association increased substantially after adjusting for FM (*β*_IVW_ = 0.07, 95%CIs = 0.05 ~ 0.09, *P* value = 2.51 × 10^−13^), BFP (*β*_IVW_ = 0.04, 95%CIs = 0.02 ~ 0.05, *P* value = 1.06 × 10^−5^), BMI (*β*_IVW_ = 0.07, 95%CIs = 0.05 ~ 0.08, *P* value = 1.92 × 10^−16^), or BW (*β*_IVW_ = 0.13, 95%CIs = 0.09 ~ 0.17, *P* value = 6.33 × 10^−11^, Fig. 5 and Additional file 2: Tables S13–S18).

### Sensitivity analysis

Univariable MR results using effect sizes from the BMI-GWAS (2015) remained consistent with our results. MR-Clust analysis also showed consistent patterns, with clusters 1–3 maintaining positive effects on eBMD and clusters 4–6 maintaining negative effects, confirming the robustness of our findings. Tissue-partitioned MR results preserved similar effect directions but showed reduced statistical significance for independent skeletal muscle-tissue instrumented BMI effects, likely reflecting decreased statistical power, suggesting the need for validation with larger independent datasets in future (Additional file 2: Tables S19–S26).

## Discussion

Leveraging the hitherto largest genome-wide genetic summary data and diverse MR methodologies, this study conducted a comprehensive re-evaluation of the link between BMI and eBMD, providing novel insights into the underlying distinct causal pathways to further understand the obesity paradox. After validating the protective association between genetically predicted BMI and eBMD, we first identified evidence supporting potential distinct causal pathways contributing to the observed total effect, with some exerting a protective effect on eBMD while others leading to its deterioration. Furthermore, we identified preliminary evidence suggesting that skeletal muscle-related pathway may play a more predominant role than adipose-related pathways in driving the protective effect of BMI on eBMD. This observation was supported by the independent association between genetically predicted LM and eBMD, especially after accounting for the effects of adiposity-related traits; however, further validation is still warranted.

While BMI is widely used due to its ease of measurement, its high-heterogeneous nature contributes to the complex role of BMI in bone mass, which is largely supported by the heterogeneity determined by Cochran’s *Q* statistic in our and previous MR studies [11]. To systematically analyze the heterogeneity and pleiotropy of BMI-associated SNPs and their associations with eBMD, we employed MR-Clust to successfully divide BMI-associated SNPs into clusters which exhibited either positive (clusters 1–3) or negative (clusters 4–6) associations with eBMD, significantly attenuating the observed heterogeneity within each cluster. Enrichment analysis further elucidated distinct pathways underlying the positive and negative clusters. SNPs associated with positive associations were predominantly enriched in pathways related to neuronal processes, whereas those linked to negative associations were more prominently associated with cell growth and developmental regulation pathways. The complexity between obesity and bone mass has also been supported by previous observational studies, including multiple pathways such as mechanical loading, adiposity types, and fat tissue distribution. These diverse factors likely operate through distinct regulatory mechanisms, such as the Wnt/β-catenin signaling pathway activation, excessive estrogen production, pro-inflammatory cytokines secretion, and competitively osteoblast generation inhabitation, ultimately influencing bone mass in a multifaceted manner [54, 55]. Leveraging the MR-Clust method, we underscore the multifaceted nature of the relationship from another aspect, revealing a complex interplay of diverse physiological and molecular mechanisms, thus reemphasizing the inaccurateness of using BMI as a general indicator of obesity in osteoporosis prevention.

By considering the effects of distinct subcomponents of BMI, such as muscle and fat, our work provides preliminary insights into the protective association between BMI and BMD from a tissue-specific perspective. Our findings suggest a potentially more predominant role of skeletal muscle-related pathways over adipose-related pathways in this association, though further validation is needed. In fact, the positive correlation between muscle and BMD has been consistently observed in many studies. For instance, a meta-analysis of 44 studies found a strong protective association between LM and BMD [56]. Another meta-analysis of 26 studies demonstrated that older adults with sarcopenic obesity had lower femoral neck areal BMD compared to those with obesity alone [57]. Several potential mechanisms have been proposed to explain such a relationship. Mechanical stress generated from muscle weight (a major part of body weight) and muscle contraction activates the Wnt/β-catenin signaling pathway, which enhances bone formation [58, 59]. Muscle contraction and relaxation can also improve blood flow to bone tissues, promoting nutrient delivery and stimulating bone metabolism [60]. Moreover, skeletal muscle secretes a variety of different growth factors (e.g., IGF-1, FGE-2) and cytokines (e.g., IL-15, IL-8) that play a positive role in inducing osteoblast activation and promoting bone metabolism [58, 61]. Compared with muscle, the relationship between adiposity and bone mass is more intricate. On one hand, excess adipose tissue can competitively inhibit osteoblast generation and promote osteoclast differentiation and bone resorption through pro-inflammatory pathways [16]. On the other hand, adipose tissue can also protect bone metabolism through the estrogen pathway and an increased mechanical loading [17, 59]. Additionally, fat deposition in different body parts may have distinct metabolic effects. Previous studies have reported a differential effect of fat in the lower (beneficial effect) and upper (detrimental effect) parts of the body on cardiovascular metabolism, primarily attributed to an increased susceptibility to adipocyte dysfunction and an elevated inflammatory response in upper-body adipose tissue, especially the visceral adipose tissue [62–64]. Although several MR studies have evaluated various central obesity traits and BMD, the results remain inconsistent [12, 65, 66] and no study has specifically focused on the role of fat distribution in eBMD. Leveraging eQTL data of visceral adipose and subcutaneous adipose tissue, we tried to figure out whether visceral adipose and subcutaneous adipose have distinct impacts on BMD. However, no significant result was observed possibly due to inadequate statistical power, necessitating further investigation to fully explore this relationship.

So far, many MR studies have examined the relationship between genetically predicted BMI and bone mass, and have confirmed a protective association between BMI and eBMD [10–12]. Nevertheless, the presence of strong heterogeneity and pleiotropy in the relationship has not been adequately explored or addressed, yielding biased results due to violations of MR assumptions [67]. In contrast, extended MR approaches such as MR-Clust and tissue-partitioned MR provide innovative methodological strategies to further disaggregate the different components and biological mechanisms underlying causal associations [20–22]. MR-Clust has been successfully applied to evaluate the distinct causal associations of adiposity and omega-3 fatty acids on type 2 diabetes [68, 69], while tissue-partitioned MR has revealed divergent underlying pathways for BMI with site-specific cancers risk and cardiometabolic phenotypes [21, 22]. Our study is the first to apply these extended MR approaches in investigating the “BMI-eBMD” link, uncovering potential divergent causal pathways, and providing promising insights into biological mechanisms. However, it should be emphasized that the estimates obtained from tissue-partitioned MR analysis should not be interpreted as directly representing causal associations, unlike in traditional MR studies. Instead, this approach serves as a tool to explore how genetic instruments associated with different tissue-related pathways of a certain trait contribute to the overall association, as suggested in the original study [21, 22].

When utilizing body composition phenotypes as supplements, we highlight an independent association between genetically predicted LM and eBMD, especially after adjusting for different adiposity phenotypes, thereby corroborating the findings of tissue-partitioned MR from another aspect. Two previous two-sample univariable MR study investigated the causal associations between muscle-related phenotypes and BMD at multiple sites [70, 71]. Nevertheless, utilizing a limited number of IVs (both *N*_IVs_ = ~ 500), both of the two study only identified a significant positive association between LM and lumbar spine BMD (*P* value < 0.05), with no significant associations for other BMD measures. With a nearly two-fold increase in the numbers of IVs (*N*_IVs_ = ~ 1000), which substantially improves statistical power, our MR analysis found evidence supporting a protective association between genetically predicted LM and eBMD. Moreover, both the magnitude and significance of this protective association were largely increased after adjusting for the four traits representing adiposity- and obesity-related characteristics, highlighting the independent role of muscle in enhancing bone mass.

Several limitations of our study need to be acknowledged. First, although eBMD is characterized by its significant hereditary component and the largest available GWAS summary dataset, our focus solely on eBMD as an osteoporosis proxy might limit the generalizability of our findings to BMD at other body sites. Future investigations are needed to explore the effects of BMI on site-specific BMD. Second, while we provided a new perspective on the obesity paradox between BMI and BMD for the first time by using a tissue-partitioned MR framework, the sample size of the eQTL data in our analysis was less than 1000 individuals due to data limitations, which may affect the robustness of the results and lead to smaller *F*-statistics (< 10, Additional file 2: Table S11) in multivariable analysis in the tissue-partitioned MR [72]. Despite the small sample size, we still found several lines of suggestive evidence, indicating that future research should validate the findings using larger datasets, when available. Moreover, while our analysis solely focused on skeletal muscle and adipose tissues, this does not preclude other tissues and pathways from being involved in the BMI-eBMD relationship. Third, UKB participant overlap across exposure and outcome datasets could bias our estimates [73]. Although two-sample MR can be safely used with large biobank data [74], and we utilized strong instruments and validated our main findings with non-overlapping GWAS data, future validation using larger non-overlapping datasets would nonetheless be beneficial. Finally, the prevalence of osteoporosis differs significantly between men and women [75]; however, due to data limitations, gender differences were not explored in our study. Further research is warranted to address this gap when sex-specific GWAS and eQTL data become available.

## Conclusions

In conclusion, our study sheds light on the intricate relationship between obesity and bone health, highlighting divergent causal pathways underlying the association between BMI and eBMD. Our work provides preliminary insights into the role of muscle in influencing eBMD, although further validation is needed. These findings emphasize the potential importance of precision obesity management, such as body composition measurements, over merely a general indicator as BMI in future public health strategies for osteoporosis prevention and prediction.

## Supplementary Information

Additional file 1: STROBE-MR checklists.

Additional file 2: Tables S1–S26: Table S1 Details of GWAS summary data. Table S2 Characteristics of instrumental variables selected for BMI. Table S3 Cochran’s Q for the genetically predicted effects of BMI on eBMD. Table S4 Characteristics of index SNPs in each cluster obtained from MR-Clust analysis. Table S5 Causal estimates of univariable MR analysis of BMI on eBMD using IVs in substantive clusters obtained from MR-Clust. Table S6 Biological pathways for SNPs in clusters with different effects identified by MR-Clust. Table S7 Characteristics of index SNPs identified by colocalization analysis incorporated into the skeletal muscle-tissue instrumented BMI. Table S8 Characteristics of index SNPs identified by colocalization analysis incorporated into the visceral adipose-tissue instrumented BMI. Table S9 Characteristics of index SNPs identified by colocalization analysis incorporated into the subcutaneous adipose-tissue instrumented BMI. Table S10 The average effect sizes of tissue-partitioned instruments. Table S11 Conditional F-statistics for tissue-specific instrumented BMI in multivariable MR analysis. Table S12 Results of tissue-partitioned MR using IVs obtained from a more relaxed threshold of PPA4 > 0.7. Table S13 Characteristics of index SNPs associated with LM or FM in multivariable MR analysis. Table S14 Characteristics of index SNPs associated with LM or BFP in multivariable MR analysis. Table S15 Characteristics of index SNPs associated with LM or BMI in multivariable MR analysis. Table S16 Characteristics of index SNPs associated with LM or BW in multivariable MR analysis. Table S17 Conditional F-statistics for IVs of body composition phenotypes in multivariable MR analysis. Table S18 Causal estimates of univariable and multivariable MR analysis of body composition phenotypes other than LM on eBMD. Table S19 Characteristics of instrumental variables selected for BMI with effect sizes from BMI-GWAS 2015. Table S20 Sensitivity analysis of univariable Mendelian randomization analysis conducted for BMI and eBMD. Table S21 Characteristics of index SNPs in each cluster obtained from MR-Clust analysis with effect sizes from BMI-GWAS 2015. Table S22 Sensitivity analysis of the effect of genetically predicted BMI on eBMD using IVs in substantive clusters obtained by MR-Clust. Table S23 Characteristics of index SNPs identified by colocalization analysis incorporated into the skeletal muscle-tissue instrumented BMI with effect sizes from BMI-GWAS 2015. Table S24 Characteristics of index SNPs identified by colocalization analysis incorporated into the visceral adipose-tissue instrumented BMI with effect sizes from BMI-GWAS 2015. Table S25 Characteristics of index SNPs identified by colocalization analysis incorporated into the subcutaneous adipose-tissue instrumented BMI with effect sizes from BMI-GWAS 2015. Table S26 Sensitivity analysis of tissue-partitioned MR conducted for BMI and eBMD.

## Data Availability

All data utilized in this study are publicly accessible. GWAS summary data are available from the previously original studies (GWAS summary data of BMI: https://www.zenodo.org/records/1251813, GWAS summary data of eBMD: https://www.gefos.org/?q=content/data-release-2018, GWAS summary data of LM: https://www.gwas.mrcieu.ac.uk/datasets/ukb-a-266/, GWAS summary data of FM: https://www.gwas.mrcieu.ac.uk/datasets/ukb-b-19393/, GWAS summary data of BFP: https://www.ftp.ebi.ac.uk/pub/databases/gwas/summary_statistics/GCST007001-GCST008000/GCST007064/, and GWAS summary data of BW: https://www.ftp.ebi.ac.uk/pub/databases/gwas/summary_statistics/GCST90018001-GCST90019000/GCST90018949/). The data of eQTL are available at the Summary Mendelian Randomization website (https://www.cnsgenomics.com/software/smr/), which were mapped to hg19 genome build using the GRCh37 reference assembly. This study does not generate any original code. The software, R packages, and other resources used in this study are accessible at: NHGRI-EBI GWAS Catalog, https://www.ebi.ac.uk/gwas/, IEU Open GWAS Project https://www.gwas.mrcieu.ac.uk/, PLINK, https://www.cog-genomics.org/plink/1.9/, LDlinkR, https://www.github.com/CBIIT/LDlinkR, TwoSampleMR, https://www.mrcieu.github.io/TwoSampleMR/, MR-PRESSO, https://www.github.com/rondolab/MR-PRESSO, MR-Clust, https://github.com/cnfoley/mrclust, VEP: https://www.grch37.ensembl.org/info/docs/tools/vep/index.html, WebGestalt: https://www.webgestalt.org/, MVMR, https://www.github.com/WSpiller/MVMR, MendelianRandomization, https://www.github.com/cran/MendelianRandomization, Coloc, https://www.chr1swallace.github.io/coloc/. Any additional information required to reanalyze the data reported in this study is available from the corresponding author upon reasonable request.

## Abbreviations

BBJ: Biobank Japan
BFP: Body fat percentage
BMD: Bone mineral density
BMI: Body mass index
BW: Body weight
CI: Confidence interval
eBMD: Estimated bone mineral density
eQTL: Expression quantitative trait loci
FM: Fat mass
GIANT: Genetic Investigation of Anthropometric Traits
GO: Gene Ontology
GTEx: Genotype-Tissue Expression
GWAS: Genome-wide association study
IV: Instrumental variable
IVW: Inverse variance weighted
KEGG: Kyoto Encyclopedia of Genes and Genomes
LD: Linkage disequilibrium
LM: Lean mass
MR: Mendelian randomization
MR-PRESSO: Mendelian Randomization Pleiotropy Residual Sum and Outlier
PPH: Posterior Probability of Hypothesis
SD: Standard deviation
SNP: Single nucleotide polymorphism
UKB: UK Biobank
VEP: Ensembl Variant Effect Predictor
